# Functional implication of ubiquitinating and deubiquitinating mechanisms in TDP-43 proteinopathies

**DOI:** 10.3389/fcell.2022.931968

**Published:** 2022-09-09

**Authors:** Non-Nuoc Tran, Byung-Hoon Lee

**Affiliations:** ^1^ Department of New Biology, Daegu-Gyeongbuk Institute of Science and Technology (DGIST), Daegu, South Korea; ^2^ Department of New Biology Research Center (NBRC), Daegu-Gyeongbuk Institute of Science and Technology (DGIST), Daegu, South Korea

**Keywords:** ubiquitinating enzyme, deubiquitinating enzyme, ubiquitin-proteasome system, TDP-43, protein quality control, proteinopathy, frontotemporal lobar degeneration, amyotrophic lateral sclerosis

## Abstract

Amyotrophic lateral sclerosis (ALS) is a fatal neurodegenerative disease in which motor neurons in spinal cord and motor cortex are progressively lost. About 15% cases of ALS also develop the frontotemporal dementia (FTD), in which the frontotemporal lobar degeneration (FTLD) occurs in the frontal and temporal lobes of the brain. Among the pathologic commonalities in ALS and FTD is ubiquitin-positive cytoplasmic aggregation of TDP-43 that may reflect both its loss-of-function and gain-of-toxicity from proteostasis impairment. Deep understanding of how protein quality control mechanisms regulate TDP-43 proteinopathies still remains elusive. Recently, a growing body of evidence indicates that ubiquitinating and deubiquitinating pathways are critically engaged in the fate decision of aberrant or pathological TDP-43 proteins. E3 ubiquitin ligases coupled with deubiquitinating enzymes may influence the TDP-43-associated proteotoxicity through diverse events, such as protein stability, translocation, and stress granule or inclusion formation. In this article, we recapitulate our current understanding of how ubiquitinating and deubiquitinating mechanisms can modulate TDP-43 protein quality and its pathogenic nature, thus shedding light on developing targeted therapies for ALS and FTD by harnessing protein degradation machinery.

## Introduction

**FIGURE 1:**
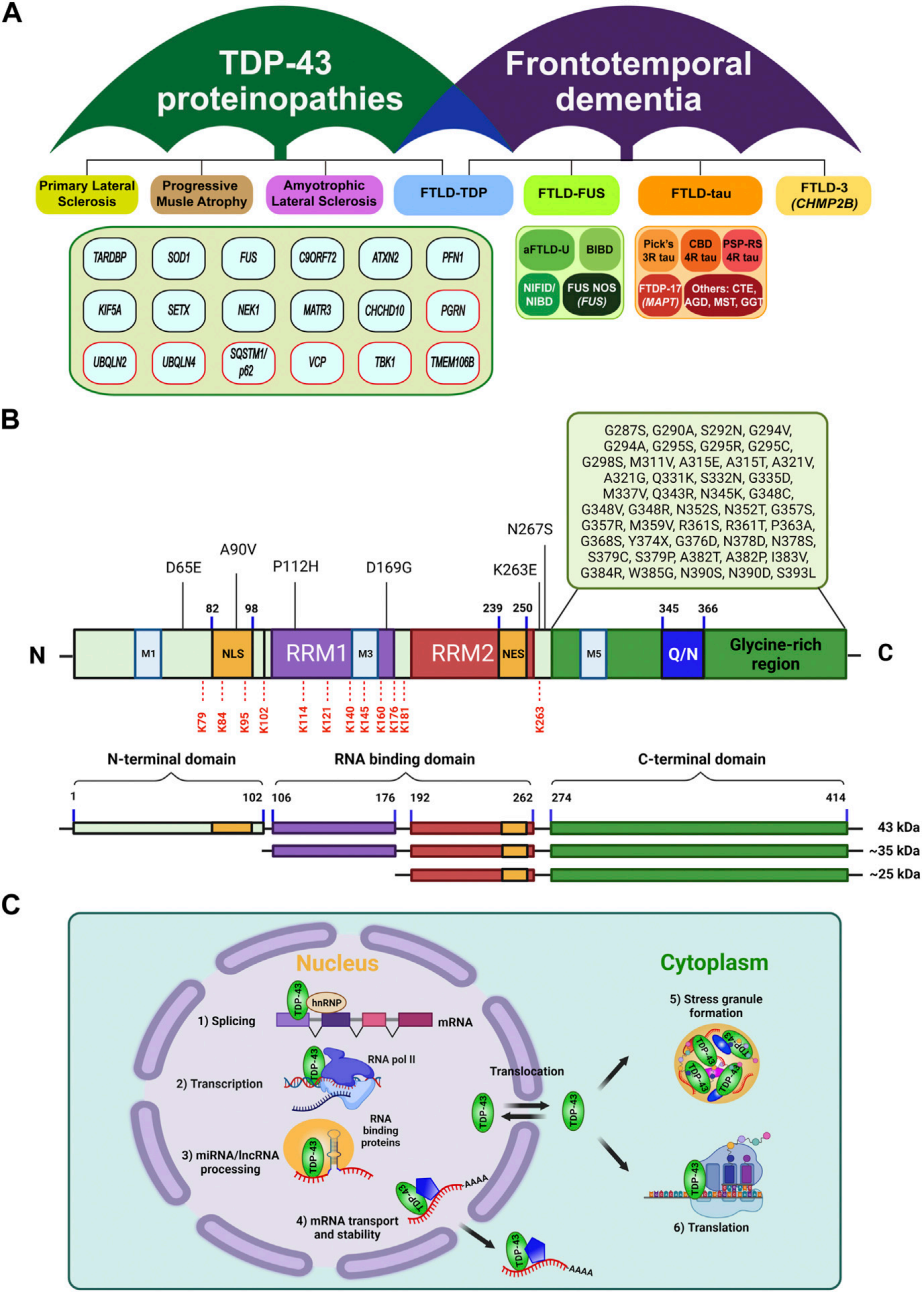
Classification of TDP-43 proteinopathies, and its structural features and general functions. **(A)** Umbrella diagram of TDP-43 proteinopathies and frontotemporal lobar degeneration (FTLD) that are classified by neuropathy types and causal genetic factors or pathological signature factors (modified from Prasad et al., 2019 and Hofmann et al., 2019). Genes in red boxes highlight the UPS or autophagy components. Note that the genetic factors under TDP-43 proteinopathies were primarily identified from ALS and FTLD-TDP. **(B)** Structural organization of TDP-43. TDP-43 is composed of an N-terminal domain (NTD) with a nuclear localization signal (NLS), two RNA-recognition motifs (RRM1, RRM2) with a nuclear export signal (NES) in RRM2, and a glycine-rich C-terminal domain (CTD) containing a glutamine/asparagine-rich region (Q/N). Approximately 50 missense mutations were located in the glycine-rich region of CTD (Buratti, 2015; Harrison and Shorter, 2017). Under pathological conditions, the full length TDP-43 can undergo truncation into the C-terminal fragments of 35 kDa or 25 kDa via caspase or calpain (Zhang et al., 2009; Xiao et al., 2015; Berning and Walker, 2019). In addition, a number of ubiquitination sites in TDP-43 have been reported, including K79, K84, K95, K102, K114, K121, K140, K145, K160, K176, K181, and K263, which are marked in red dash lines (Kim et al., 2011; Dammer et al., 2012; Kametani et al., 2016; Hans et al., 2018). **(C)** TDP-43 is mainly localized in nucleus where it performs multiple functions relating to RNA metabolic pathways, including 1) mRNA splicing, 2) transcription, 3) miRNA biogenesis, and lncRNA processing. Also, it shuttles between nucleus and cytoplasm, where it participates in 4) & 6) mRNA transport, stability, protein translation, and 5) stress granule assembly.

Amyotrophic lateral sclerosis (ALS) is among the most prevalent and fatal neurodegenerative diseases in which motor neurons in spinal cord and motor cortex are progressively lost. Patients diagnosed with ALS suffer from the gradual respiratory dysfunction, having the mean survival time of 3–5 years due to no effective therapeutic treatment (Mejzini et al., 2019). Approximately 90–95% of patients show sporadic ALS, while only less than 10% belong to familial cases (Ling et al., 2013). Frontotemporal lobar degeneration (FTLD), the second-highest incidence rates of early-onset dementia after Alzheimer’s disease, is characterized by the cumulative neuronal loss in the frontal and temporal lobes of the brain, manifesting behavioral abnormalities, personality changes, and gradual language inability (Van Mossevelde et al., 2018; Ferrari et al., 2019). It was reported that about 15% cases of FTLD show ALS symptoms, meanwhile as much as 15% of ALS patients also develop classic features of FTLD such as cognitive impairment (Ringholz et al., 2005; Wheaton et al., 2007). Such shared modality can be explained by the fact that there is a distinctively broad but also overlapping spectrum of ALS and FTLD in terms of clinical aspects, neuropathological mechanisms, and genetic mutations (Figure 1A) (Ling et al., 2013; Ferrari et al., 2019). In fact, TAR DNA-binding protein-43 (TDP-43) was found to account for the connecting pathology of more than 90% of ALS and about 50% of FTD (i.e., FTLD-TDP) cases (Ling et al., 2013). Strikingly, ubiquitinated and hyper-phosphorylated TDP-43 was identified as a primary constituent of the mislocalized and insoluble cytoplasmic inclusions in the patient brain samples (Arai et al., 2006; Neumann et al., 2006). This observation may support the idea that the proteolytic control of TDP-43 is intimately connected to the pathomechanisms in ALS and FTD (Gao et al., 2018). However, it still remains as questions of how the TDP-43 pathogenesis upends this quality controlling protein clearance system. In this review, we briefly summarize the recent progress of ubiquitination and deubiquitination mechanisms that can modulate TDP-43 protein stability, localization and stress response, which may provide the mechanistic insights into developing proteolysis-based treatment for TDP-43 proteinopathies. Detailed physiology and pathology of TDP-43 have been extensively discussed recently (Prasad et al., 2019; Keating et al., 2022).

## Structure and function of TDP-43

A 414-amino acid TDP-43 protein contains an N-terminal domain with a nuclear localization signal (NLS), two RNA recognition motifs (RRM1 and RRM2) with a nuclear export signal (NES), mitochondria localization signals (M1-M5), and a glycine-rich C-terminal domain where multiple ALS-associated mutations were reported (Figure 1B) (Guerrero et al., 2016; Prasad et al., 2019). This feature defines the predominant localization of TDP-43 in the nucleus where it regulates RNA metabolism, including transcription, splicing, translation as well as stability (Pinarbasi et al., 2018; Weskamp and Barmada, 2018; Bjork et al., 2022), while being also capable of translocation to the cytoplasm to mediate mRNA transport (Chu et al., 2019) or stress granule formation (Khalfallah et al., 2018) (Figure 1C). Indeed, deletion or A90V mutation in the NLS promotes the insoluble cytoplasmic TDP-43 mislocalization and aggregation (Winton et al., 2008a; Winton et al., 2008b; Barmada et al., 2010). Two RRM domains and possibly their dimerization are necessary for the proper binding of TDP-43 to DNA/RNA molecules with high specificity towards TG/UG-rich sequences. Several studies indicated that mutations in these regions impair TDP-43’s RNA binding and splicing activities (Lukavsky et al., 2013; Kuo et al., 2014; Furukawa et al., 2016), confirming its critical role in RNA metabolism. Notably, most of ubiquitination sites on TDP-43 so far have been identified within or near these RRM domains (Figure 1B) (Kim et al., 2011; Dammer et al., 2012; Kametani et al., 2016; Hans et al., 2018). The C-terminal domain (CTD) represents a highly disordered and low-complexity domain (LCD) which consists of a glycine-rich region separated by a glutamine and asparagine (Q/N)-enriched segment (Figure 1B). The CTD has been of great interest due to its intrinsic aggregation-prone property like the prion-like domain (King et al., 2012) which apparently contributes to TDP-43-induced pathological inclusions and neurotoxicity in ALS or FTLD-TDP (Afroz et al., 2017; Santamaria et al., 2017). Furthermore, most of ALS-causing mutations, cytotoxic truncated forms, and phosphorylation sites have been associated with the CTD of TDP-43 (Figure 1B) (Hasegawa et al., 2008; Pesiridis et al., 2009; Zhang et al., 2009; Xiao et al., 2015; Berning and Walker, 2019). The N-terminal domain (NTD) also exhibits its own oligomerization or aggregation propensity (Tsoi et al., 2017), and intriguingly, the part of NTD adopts a novel ubiquitin-like fold that can bind to ssDNA (Qin et al., 2014). It should be further investigated whether this structural element can also provide extra avidity for forming aggregates or association with ubiquitin binding proteins under pathological conditions. Overall, these structural features endow multi-dimensional regulation of TDP-43 protein, in which ubiquitin signaling may also serve as a critical surveillance system for its proper functioning through proteostasis networks.

## Pathological mechanisms of TDP-43

Failure of TDP-43 regulation culminates in the fatal outcome of neuronal defect obviously because its pleiotropic nature may represent the sum from any of proteostasis, RNA homeostasis, liquid condensate homeostasis (as in stress granule), and organelle homeostasis (as in mitochondria) (Keating et al., 2022). Under physiological conditions, TDP-43 predominantly exerts nuclear functions in RNA-related processes but also shuttles between nucleus and cytoplasm to participate in stress granule formation and mRNA translation (Figure 1C). By contrast, under pathological mutations or stressors, nuclear depletion of TDP-43 seems to precede, and then entails its cytoplasmic mislocalization, aggregation, and inclusion formation (Tomé et al., 2020). Also, the pathological hallmark of TDP-43 proteinopathies highlights the aberrant deposition of ubiquitinated and hyper-phosphorylated TDP-43 in the cytoplasmic inclusions, which indicates that the proteolytic control of TDP-43 must have been severely compromised (Klaips et al., 2018). Therefore, for TDP-43-induced neurotoxicity, TDP-43 loss-of-function likely occurs due to its nuclear depletion accompanying cytoplasmic accumulation, which in turn progressively drives the pathogenic aggregation and deposition of insoluble TDP-43 inclusions (Lee et al., 2012). Probably, such dramatic changes of the molecular signature inevitably drive the progression of TDP-43 pathogenesis by altering or disturbing its *bona fide* protein (and also RNA) interaction networks. Above certain threshold that can be held by protein quality control mechanisms–such as ubiquitin-proteasome system (UPS), heat shock response, or autophagy-lysosomal degradation pathway, TDP-43 proteopathies can become runaway or even aggravated by faulty proteostasis pathways. Moreover, aberrant accumulation of pathological TDP-43 species sequesters the critical UPS components, directly impairs the proteasome activity, induces the accumulation of insoluble polyubiquitinated proteins, and also depletes free ubiquitin pool, thereby leading to perturbed protein and ubiquitin homeostasis (Cicardi et al., 2018; Lee et al., 2020b; Farrawell et al., 2020; Riemenschneider et al., 2022). In consistent with this view, dysregulation of TDP-43 in ALS and FTLD-TDP disrupts multiple physiological events and manifests a complex set of pathomechanisms–for example, 1) the loss-of-TDP-43 function impairs a range of its nuclear functions in RNA metabolism such as alternative or exon splicing, RNA biogenesis or stability, and polyadenylation (Yang et al., 2014; Mitra et al., 2019; Ni et al., 2021); 2) the gain-of-toxicity of TDP-43 may further exacerbate the pathogenesis by sequestering other important biomolecules and organelles through undesirable interactions (Igaz et al., 2011; Zuo et al., 2021). Accumulation of cytoplasmic TDP-43 also represses the global protein synthesis in neuroblastoma models and FTD brain samples (Russo et al., 2017; Charif et al., 2020). In addition, a growing list of evidences indicate that TDP-43-induced neuroinflammation and innate immune responses are critically associated with the TDP-43 pathogenesis via NF-κβ/p65, cGAS-STING, NLRP3 inflammasome and PTP1B pathways (Zhao et al., 2015; Lee et al., 2020a; Dutta et al., 2020; Yu et al., 2020; Bright et al., 2021).

So far, over 50 missense mutations in *TARDBP* gene (encoding TDP-43) have been associated with ALS and FTLD-TDP (Buratti, 2015; Harrison and Shorter, 2017), most of which being clustered within the aggregation-prone CTD region (Figure 1B). Remarkably, although *TARDBP* mutations account for only 5–10% of familial ALS and the remaining over 90% are attributable to other genes such as *C9ORF72*, *SOD1*, *FUS*, and *UBQLN2* (Kim et al., 2020), the majority of ALS cases (about 97%) also exhibit TDP-43-induced pathology (Ling et al., 2013). TDP-43 mutations were proposed to alter the protein stability or increase the propensity of mislocalization or aggregation. The reported half-lives of TDP-43 wild-type and mutants have been somewhat inconsistent among the literatures: while some studies observed the more prolonged turnover rate of TDP-43 mutants (Ling et al., 2010; Watanabe et al., 2013; Austin et al., 2014), others reported the faster degradation of the mutants and the C-terminal truncated forms (Araki et al., 2014; Scotter et al., 2014). Disease-associated TDP-43 mutants (e.g., G376D, G335D, G343R, A315T, and M337V) were also reported to increase the cytoplasmic TDP-43 mislocalization and stress granule or inclusion formation, which may become deleterious to neuronal cells (Jiang et al., 2016; Mitsuzawa et al., 2018; Ding et al., 2021).

## Ubiquitination and deubiquitination events in the quality control and fate decision of TDP-43

### Ubiquitination and TDP-43

Ubiquitination is a cascade of ATP-dependent conjugation reaction that attaches the ubiquitin tag on the target substrate by the sequential cooperation of E1 (ubiquitin activating enzyme), E2 (ubiquitin conjugating enzyme), and E3 (ubiquitin ligase) enzymes (Hershko and Ciechanover, 1998). By generating diverse configurations and linkage types of chains, the ubiquitination pathways are critically involved in both proteolytic (as in UPS and autophagy-lysosomal degradation) and non-proteolytic processes (as in signal transduction and membrane trafficking) (Yau and Rape, 2016; Pohl and Dikic, 2019). Strikingly, ubiquitin-positive inclusion represents one of the pathological hallmarks of many types of neurodegenerative diseases (Schmidt et al., 2021), suggesting that the protein quality control mechanisms must have been dysfunctional in general. Likewise, ubiquitinated TDP-43 has been also enriched in the ALS and FTD brain inclusions (Arai et al., 2006; Neumann et al., 2006).

**FIGURE 2:**
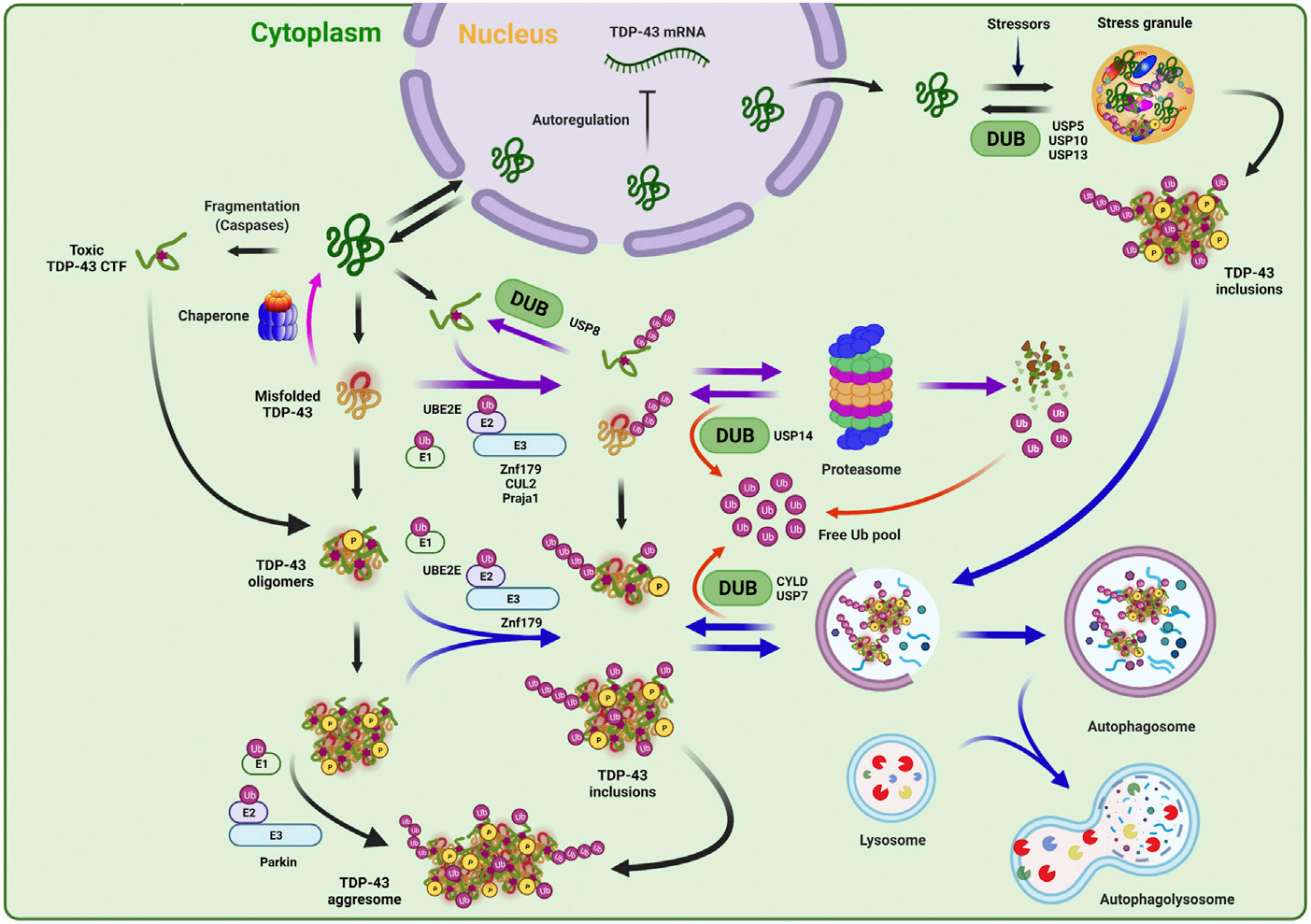
Ubiquitination and deubiquitination mechanisms in TDP-43 regulation. TDP-43 protein level can be tight regulated by multi-dimensional proteostasis surveillance systems. In the normal conditions, the TDP-43 amount in the cell is maintained via a negative feedback loop mechanism in which TDP-43 autoregulates and suppresses its own mRNA expression. Under the pathological conditions, TDP-43 translocates from nucleus to cytoplasm to be accumulated, and can be turned into various pathological species, such as misfolded, oligomeric, or truncated toxic C-terminal TDP-43 fragment. Under the prolonged stress conditions, TDP-43 also can be recruited into aberrant stress granules, forming toxic aggregates or insoluble inclusions. TDP-43 can be refolded by chaperones or recognized and eliminated by ubiquitin-proteasome system and autophagy pathway. E2 (UBE2E) and E3 (Parkin, Znf179, CUL2, and Praja 1) enzymes, which have been reported to regulate the quality control or fate decision of TDP-43, are indicated. Note that depending on the ubiquitination events by given enzymes, the ubiquitin conjugates are doomed to be degraded by proteasome or autophagy, or rather to form the pathological aggregates or inclusions. This ubiquitination process can be reversed by deubiquitinating enzymes (DUBs) (USP8, USP14, USP7, and CYLD), thereby regulating TDP-43 stability, editing ubiquitin architecture, and maintaining free ubiquitin pool. DUBs that are involved in stress granule dynamics (USP5, USP10, and USP13) were also indicated.

Indeed, the maintenance of TDP-43 proteostasis seems to be critical for its proper functioning because either upregulation or downregulation of TDP-43 can drive the neurotoxicity in various model systems (Ash et al., 2010; Li et al., 2010; Miguel et al., 2011; Zhang et al., 2012; Ling et al., 2013). TDP-43 level is also tightly regulated through autoregulation for which TDP-43 protein represses its own mRNA translation by binding to the 3’ untranslated region (Figure 2) (Ayala et al., 2011). The coordinated operation of UPS, chaperones, and autophagy likely influences the balanced turnover of TDP-43 protein. Thus, it is not surprising that a number of ALS- and FTLD-TDP-associated mutations have been identified in the UPS or autophagic components, such as *UBQLN*, *VCP*, *p62*, and *OPTN* (Figure 1A) (Medinas et al., 2017; Keating et al., 2022). It was suggested that the soluble TDP-43 (either wild-type or the pathologic forms) may undergo efficient degradation by the UPS, but once the cytotoxic oligomers or aggregates being formed, they are preferentially cleared by autophagy-lysosomal degradation pathway (Scotter et al., 2014).

UBE2E3 was identified to be an E2 ubiquitin-conjugating enzyme that is involved in the ubiquitination process of TDP-43. This E2 enzyme physically interacts with TDP-43 and promotes its ubiquitination and insolubility (Figure 2) (Hans et al., 2014). A few of E3 ubiquitin ligase enzymes have been also reported to participate in the regulation of TDP-43 proteinopathies (Figure 2). Hebron et al. have reported that the E3 ligase Parkin and TDP-43 form a multi-protein complex with HDAC6, and this interaction facilitates the cytoplasmic accumulation of TDP-43 and also mediates its K48- and K63-linked polyubiquitination. Interestingly, this Parkin-induced TDP-43 ubiquitination only promotes its cytoplasmic mislocalization and inclusion formation without any indication of the proteolytic clearance (Hebron et al., 2013). In 2016, Uchida et al. revealed that VHL/CUL2 E3 ligase ubiquitinates and enhances the degradation of C-terminal TDP-43 fragments of 35 and 25 kDa by favorably recognizing misfolded form of TDP-43 at E246 site in RRM2 domain. Unexpectedly, excess VHL instead stabilizes TDP-43 and enhances its inclusion formation, suggesting the importance of balanced proteolytic control by VHL/CUL2 complex in ALS (Uchida et al., 2016). Another RING E3 ubiquitin ligase, Znf179 was identified to interact with and polyubiquitinate TDP-43 *in vitro* and *in vivo*. The Znf179-mediated ubiquitination enhances TDP-43 protein degradation and also antagonizes its cytoplasmic mislocalization and insoluble aggregation. Conversely, Znf179-knockout in mouse brain accumulates insoluble and cytoplasmic TDP-43 inclusions in neuronal tissues (Lee et al., 2018). Recently, one study also reported that the Praja 1 E3 ligase exerts remarkably suppressive effects on phosphorylation and aggregation of pathological cytoplasmic TDP-43 C-terminal fragment (CTF) both *in vitro* and *in vivo*, although it was not determined whether Praja 1 is able to ubiquitinate TDP-43 (Watabe et al., 2020). Furthermore, Praja 1 was also found to interact with E2 ubiquitin-conjugating enzyme UBE2E3, implying that the pair of Praja1/UBE2E3 may induce the ubiquitination of TDP-43 against the pathogenic process, contrary to the outcome from only UBE2E3-mediated ubiquitination (Hans et al., 2014; Watabe et al., 2020). Overall, aberrant ubiquitination and defective degradation of TDP-43 may underly the pathological mechanisms in TDP-43 proteopathies, in which reduced proteasomal and autophagic activities along with augmented proteolysis demand by TDP-43 accumulation render the vicious cycle into progressively aggravating the pathogenesis (Figure 2).

### Deubiquitination and TDP-43

Deubiquitinating enzymes (DUBs) can exclusively reverse the ubiquitination process and thus are capable of stabilizing the target proteins. Besides, DUBs also play essential roles in ubiquitin recycling and ubiquitin chain editing, which may lead to altered subcellular localization of substrates and various responses to cellular signaling (Komander et al., 2009; Clague et al., 2019). Although DUBs may represent potential drug targets for modulating TDP-43 protein turnover, only a few have been implicated in TDP-43 regulation or stress granule formation including USP5, USP7, USP8, USP10, USP13, USP14, and CYLD (Figure 2) (Lee et al., 2010; Hans et al., 2014; Xie et al., 2018; Dobson-Stone et al., 2020; Zhang et al., 2020).

USP14, one of the major DUBs on the proteasome, can deubiquitinate and stabilize the protein substrates that are ubiquitinated at multiple sites (Lee et al., 2010; Lee et al., 2016). Given the remarkable nature of USP14–i.e., highly activated only when bound to the proteasome and potent suppression of substrate degradation, small-molecule USP14 inhibitors (e.g., IU1 series) have been also developed as drug-like molecules (Lee et al., 2010; Boselli et al., 2017). In fact, overexpression of USP14 in MEF cells substantially stabilized the protein level of TDP-43, whereas USP14 inhibitor treatment remarkably enhanced the turnover of neurotoxic substrates including TDP-43 (Lee et al., 2010; Boselli et al., 2017). USP8/UBPY was identified to modulate TDP-43-induced neurotoxicity (Hans et al., 2014). This DUB was previously known to regulate endocytosis via interactions with ESCRT-associated protein components such as EGFRs or STAM (Clague and Urbé, 2006). By employing yeast two-hybrid screening and co-immunoprecipitation, Hans et al. revealed that UBPY is a novel interacting partner of pathogenic TDP-43. Deficiency of UBPY noticeably promotes TDP-43 ubiquitination but the resulting conjugates may serve as unfavorable proteasomal substrates or just overburden the proteasome. Consequently, UBPY-silenced TDP-43 ubiquitination accelerates insoluble TDP-43 accumulation and aggregation as well as neurotoxicity *in vivo* fly model. Thus, in this case, UBPY may serve as a protective factor against TDP-43-induced neurotoxicity (Hans et al., 2014). DUBs may be also involved in autophagy-lysosome pathway for TDP-43 regulation. Recently, Dobson-Stone et al. reported that a missense mutation in CYLD (CYLD^M719V^), which was genetically identified from ALS and FTLD disease locus, exhibits significantly elevated K63-linkage specific deubiquitinase activity and caused the impairment in autophagosome-lysosome fusion pathway. This CYLD^M719V^ mutant also increased cytoplasmic mislocalization of TDP-43 and reduced the axonal length (Dobson-Stone et al., 2020). Similarly, USP7 was identified as a negative regulator of autophagy via deubiquitination of NEDD4L, an E3 ubiquitin ligase, and inactivation of TGFβ-SMAD pathway. Pharmacological inhibition or genetic suppression of USP7 significantly reduced the levels of misfolded SOD1 and TDP-43 (wild-type and Q331K), leading to attenuation of mutant SOD1 or TDP-43-induced neurotoxicity in fruit flies (Zhang et al., 2020).

Aberrant stress granule dynamics represent another pathological feature of TDP-43 proteinopathy (Hallegger et al., 2021). Notably, a handful of DUBs have been involved in stress granule formation or liquid-liquid phase separation (LLPS). USP10 was found to be localized in stress granule and also interacts with TDP-43 and G3BP1, a well-known stress granule marker (Soncini et al., 2001; Freibaum et al., 2010). USP10 can suppress the formation of aberrant cytoplasmic TDP-43/TDP-35 aggregates by enhancing the clearance of stress granules in neuronal cells (Takahashi et al., 2022). Two other DUBs, USP5 and USP13, can be also recruited to heat-induced stress granules and regulate their assembly or disassembly (Figure 2) (Xie et al., 2018). Due to the apparent difference in their mode of activities, USP5 regulates the stress granule dynamics by preferentially cleaving unanchored ubiquitin chains, whereas USP13 does so through deubiquitination of protein-conjugated ubiquitin chains (Xie et al., 2018). However, it should be further examined whether USP5 or USP13 could also functionally regulate TDP-43-positive stress granules in ALS and FTD models.

## Conclusion

While ubiquitin-enriched cytoplasmic inclusion of TDP-43 is considered to be the proteopathic signature of ALS and FTD, the pathophysiological meaning of the ubiquitination has been a long-standing question. Dramatic alteration of TDP-43 localization, stability, and post-translational modifications under the pathological conditions may disturb its genuine functional interaction networks by losing nuclear functions or gaining cytoplasmic toxicity. On top of TDP-43’s cytoplasmic mislocalization and aggregation-prone property, the initial pathogenic cue may trigger ‘butterfly effect’ and eventually the TDP-43 proteinopathy would prevail. Reduced proteasomal or autophagic activity and free ubiquitin pool depletion will badly influence the quality control of TDP-43 protein, or rather aberrant or undesirable ubiquitination of TDP-43 from proteostasis failure may only further exacerbate the disease phenotype. In fact, ubiquitination-rich pathogenic inclusions or liquid condensates, if the modification is not cleared properly, may act as avid and irreversible absorbent chambers to sequester many important proteolysis components such as ubiquitin binding proteins and proteasome. In this sense, pharmacological modulation of the pathogenic TDP-43-specific ubiquitination or the protein turnover itself may provide promising therapeutic opportunities to cope with TDP-43 proteinopathies. Remodeling or editing the ubiquitin chains or fine control of TDP-43 half-life may favor its proteolysis flux or antagonize the disease progression. It should be also noted that the total depletion of TDP-43 is not a viable option because of its essentiality. Indeed, pharmacological activation of the UPS or lysosomal pathways have been shown to bear the promise for TDP-43 clearance. For example, as noted above, a small-molecule inhibitor targeting proteasome-bound USP14 was reported to accelerate TDP-43 turnover by enhancing the proteasome activity (Lee et al., 2010). Similarly, forskolin-activated cAMP-PKA pathway increased proteasome function and remarkably reduced the levels of TDP-43 WT and its pathological mutants (Lokireddy et al., 2015). In addition, rapamycin, which works as an autophagic activator, strongly decreased the pathogenic TDP-43 species and attenuated TDP-43-induced neurotoxicity in ALS and FTD models (Cheng et al., 2015; Lattante et al., 2015). Of note, recently emerging targeted protein degradation or proteolysis targeting chimera (PROTAC) approaches have been showing great success in induced proteolysis of the undruggable targets including neurotoxic proteins (Moon and Lee, 2018). In fact, TDP-43 PROTACs were also recently reported, and await further improvement and validation (Gao et al., 2019; Hyun and Shin, 2021). In any cases, deep understanding of ubiquitinating and deubiquitinating mechanisms in TDP-43 pathology should provide the fundamental basis to develop the proteolysis-controlling therapeutic strategies for TDP-43-associated neurodegenerative diseases.
